# A cross-cultural investigation into the dimensional structure and stability of the Barriers to Research and Utilization Scale (BARRIERS Scale)

**DOI:** 10.1186/s13104-015-1579-9

**Published:** 2015-10-24

**Authors:** Brett Williams, Ted Brown, Shane Costello

**Affiliations:** Department of Community Emergency Health and Paramedic Practice, Monash University-Peninsula Campus, McMahons Road, PO Box 527, Frankston, VIC 3199 Australia; Department of Occupational Therapy, Monash University, Victoria, Australia; Faculty of Education, Monash University, Victoria, Australia

**Keywords:** BARRIERS Scale, Validity, Psychometric properties, Research utilization, Factor structure, Dimensional stability, EBP

## Abstract

**Background:**

It is important that scales exhibit strong measurement properties including those related to the investigation of issues that impact evidence-based practice. The validity of the *Barriers to Research Utilization Scale* (BARRIERS Scale) has recently been questioned in a systematic review. This study investigated the dimensional structure and stability of the 28 item BARRIERS Scale when completed by three groups of participants from three different cross-cultural environments.

**Method:**

Data from the BARRIERS Scale completed by 696 occupational therapists from Australia (*n* = 137), Taiwan (*n* = 413), and the United Kingdom (*n* = 144) were analysed using principal components analysis, followed by Procrustes Transformation. Poorly fitting items were identified by low communalities, cross-loading, and theoretically inconsistent primary loadings, and were systematically removed until good fit was achieved. The cross-cultural stability of the component structure of the BARRIERS Scale was examined.

**Results:**

A four component, 19 item version of the BARRIERS Scale emerged that demonstrated an improved dimensional fit and stability across the three participant groups. The resulting four components were consistent with the BARRIERS Scale as originally conceptualised.

**Conclusion:**

Findings from the study suggest that the four component, 19 item version of the BARRIERS Scale is a robust and valid measure for identifying barriers to research utilization for occupational therapists in paediatric health care settings across Australia, United Kingdom, and Taiwan. The four component 19 item version of the BARRIERS Scale exhibited good dimensional structure, internal consistency, and stability.

## Background

Evidence-based practice (EBP) is considered to be a combination of recent best quality evidence, clinical expertise and analysis as well as the values, expectations and views of the patient [1]. EBP is a significant factor in assuring that practice is systematic and guided by current research and that its implementation is associated with enhanced clinical outcomes in patients [2]. Research utilization is directly linked to EBP, and involves searching for, sourcing, critiquing, integrating, translating, and applying best evidence into daily professional practice.

Health professionals value EBP and research utilization, however, its implementation is often obstructed by factors such as time, skill, funding and access limitations. This trend has been reported in several health care fields including speech-language pathology, dietetics, physiotherapy [3], nursing [4] and occupational therapy [5, 6]. Parahoo [7] identified that increased EBP and continual developments in health care tertiary education were correlated with recognizing barriers to research utilization. This recognition of barriers was said to inform the most appropriate interventions, which subsequently would increase the implementation and integration of research findings into clinical practice [8], and result in enhanced patient outcomes [9]. Differences in barriers perceived in different clinical settings, cultural contexts, and health care professions further supports the importance of distinguishing barriers to research utilization [3]. Therefore having access to a valid scale that explores barriers to EBP and research utilization is timely and significant.

### Development of the BARRIERS Scale

The *Barriers to Research and Utilization Scale*, commonly known as the BARRIERS Scale, was developed in 1991 by Funk, Champagne, Wiese and Tornquist to measure barriers to research utilization as experienced by nurses [10]. Its development was focused on nurses’ perspectives as previously emphasis of identifying barriers was restricted mainly to the observations of educators and administrators [11].

The BARRIERS Scale is a self-report questionnaire, which was constructed initially in the United States with 1989 nurse participants, with credentials comprising of doctorates, masters, bachelor’s, diplomas and associate degrees [12]. The development of the scale was informed by literature, unofficial testimonies by nurses, as well as the Conduct and Utilization of Research in Nursing (CURN) questionnaire [13]. A total of 29 items were included in the initial version of the BARRIERS Scale, however the item ‘the amount of research information is overwhelming’ was found incompatible with scale’s factor structure after a factor analysis was completed and consequently this item was deleted from the scale [14].

The BARRIERS Scale is comprised of 28 items that quantify a respondent’s perceived barriers that negatively impact research utilization and EBP. Respondents rate these barriers using a four-point Likert scale and an additional “no opinion” option. Confirmatory factor analysis and Roger’s Model of Diffusion of Innovations [15] were used to identify and inform four barrier factors: (1) *characteristics of the adopter*; competency, ideals, awareness and receptiveness to research (eight items), (2) *characteristics of the organization*; practice setting (eight items), (3) *characteristics of the innovation/research*; attributes of the research (six items), and (4) *characteristics of communication*; ease of application, accessibility, and comprehension of the research (six items) [16].

Administration and application of the BARRIERS Scale has been broad across the nursing profession. A variety of different sample groups in the nursing sector have been surveyed including Pediatric Nurses, Registered Nurses, Breast Cancer Nurses, Clinical Administrators, and a multitude of practice contexts have been incorporated including palliative care, community settings, hospitals, surgical and medical practice and mental health [17]. Other specialised nursing fields have also been administered the scale, including peri-anaesthesia nurses [18] and nurses working in intensive or critical care domains [19]. Educators have completed the scale in both acute and intensive care nursing settings [20]. The BARRIERS Scale has also been completed by other health professionals including occupational therapists, physiotherapists, dieticians, and speech language pathologists [21, 22].

### Construct validity and dimensionality of the BARRIERS Scale

Much of the research involving the BARRIERS Scale has relied on rank ordering of individual items to determine the barriers to research utilization and allow for comparison across settings and professions [12]. Underlying this approach is the assumption of equivalence of single item performance across samples (both professions and cultures), despite the evidence to suggest that single items measure have lower reliability, are more vulnerable to the effects of error, and are comparatively poor predictors compared to multiple item constructs [23]. Perhaps some of the use of rank ordering of individual items is understandable given that the BARRIERS Scale has demonstrated some difficulties in replicating the theorized factor structure across samples and professions.

During the initial development, Funk et al. tested the stability of the BARRIERS Scales by dividing a sample group into two before performing a factor analysis for each [10]. Equivalent results were achieved after comparing the two factor analyses with the analysis of the entire sample: the loadings remained the same, as did the identified four factors, minimal changes in explained variance percentages were evident (sample 1; 43.4 %, sample 2; 44.9 %), a fourth factor item did not match factors three and four, but this was resolved through the final analyses. The authors of the scale have endorsed its stability in a variety of nursing contexts, and across time, despite differences occurring in numbers ascribed to individual barriers [7].

The BARRIERS Scale’s internal consistency was examined by the scale’s authors. Cronbach’s alpha coefficients ranging between 0.65 and 0.80 were reported [10]. Factors 1–3 of the scale demonstrated reasonable levels of internal consistency with Cronbach alpha coefficients of 0.75, 0.74 and 0.70 reported. Factor 4 was documented at 0.54 [24] suggesting improvements to this factor’s internal consistency could be made.

Face and content validity has been examined by others and has been found to be appropriate given that the development of the scale utilized reports from nurses’, the CURN questionnaire and literature investigating utilization of research, additionally specialists in nursing research, nursing practice, research utilization, and an expert in quantitative measurement [14]. Bayik et al. considered the scale’s validity and reliability by evaluating culturally diverse studies that used the scale [25].

Dimensional stability plays an important role in construct validity, and as previously stated, the replicability of the BARRIERS Scale factor structure has been mixed when explored in published studies. For example, Kajermo et al. completed a systematic review of studies using the BARRIERS scale [17]. Of the 63 articles examined in the review, there were a number of issues were raised surrounding the construct validity of the scale. The dimensionality of the BARRIERS scales ranged between three and eight factor solutions using both factor analysis and confirmatory factor analysis [17]. These findings suggest further psychometric investigation needs to be undertaken, including cross-cultural appraisals.

The validity of the BARRIERS Scale has been challenged when considering its wide use across clinical practice settings and cross-cultural settings. Kajermo et al. argued that changes in health care, the discipline of nursing and patient involvement have resulted in transformations in research utilization [17]. But in spite of this, the barrier factors identified by the scale remain unchanged; the theories underpinning the scale are reflective of the late 1980′s and early 1990’s health care settings, and its initial design is the one most utilized in studies. The BARRIERS *Innovation* subscale was also argued to be inadequate as it was suggested to have been simplified [17] and not truly reflective of Rogers’ Diffusion of Innovation Model [15]. One factor identified as absent from the BARRIERS Scale was related to the speed of adoption of innovation, which comprises of “relative advantage, compatibility, complexity, trialability, and observability” [26].

A study conducted in the United Kingdom employed the BARRIERS Scale to determine whether barriers to research utilization encountered by nurses working in British contexts were captured by the factors of the BARRIERS Scale [27]. The factor model was found to be unsuited to the United Kingdom data and comparatively to results obtained by the authors in the initial studies of the scale, with poorer validity, and lower reliability. Cross-cultural language was considered to impact this, and slight revisions were made to the wording of 18 scale items, however, only slight improvements in reliability were achieved [9].

Using a principal components analysis (PCA) with Varimax Rotation, Retsas and Nolan obtained a 26 item, three-factor model that accounted for 38.9 % of the variance [28]. Using the same methodology, Retsas generated a 29 item, four factor model accounting for 46.5 % of the variance [11]. In an Australian study, Hutchinson and Johnston obtained a 27 item, four factor model that accounted for 39.2 % of the variance using PCA [24]. In an American study, Ashley obtained a 29 item, four factor solution [29]. The factor analytic studies completed by Ashley [29] and Hutchinson and Johnston [24] were similar to the factor model originally reported by Funk et al. [10] These studies shed some light on the dimensional structure of the BARRIERS Scale.

Closs and Bryar investigated the appropriateness of the BARRIERS Scale for use within the context of the United Kingdom health care system [12]. The BARRIERS Scale was sent to 4,501 nurses, with a 44.6 % response rate. Using PCA with Varimax Rotation, Closs and Bryar found a 22 item, four factor solution which explained 47.5 % of the variance [12]. The four factors were labelled *benefits*; *quality of research*; *accessibility of research*; and *resources for implementation*. These were similar, but not identical, to factors identified in the original United States study. Closs and Bryar concluded that the four factor solution they obtained was similar in principle to the United States version of the BARRIERS Scale, although fewer items were retained (22 rather than 28) [12]. This indicates that there appears to be some stability in the BARRIERS Scale dimensional structure, however, the variability in number of items retained suggests that problematic items remain.

Given the mixed results of the psychometric properties of the BARRIERS Scale among nursing cohorts, further investigation of the dimensionality is warranted with other health professional participant groups from different cross-cultural contexts. Therefore the aim of this study was to investigate the dimensional structure and stability of the 28 item BARRIERS Scale when completed by three occupational therapy cohorts from three different cross-cultural environments.

## Method

### Design

A mailed survey was administered to participants in Australia, the United Kingdom, and Taiwan. Including participants from three countries provided insights into the dimensional structure and stability of the BARRIERS Scale.

### Participants

The membership data bases from OT Australia, College of Occupational Therapists in the United Kingdom, and the Taiwan Occupational Therapy Association (TOTA), were used to invite occupational therapists who indicated that pediatrics was their clinical specialty to participate in the study. The inclusion criteria for this study were: (1) consenting to participate in the study; and (2) being a qualified occupational therapist in Australia, the United Kingdom, or Taiwan. Ethics Committee approval was obtained from Monash University, Australia; the British Association of Occupational Therapists; and the Taiwan National University Hospital prior to the commencement of the study in each respective country. The justification for including participants from three different countries was that it provided an opportunity for the dimensional structure and stability of the BARRIERS Scale to be examined when completed by respondents from three different cross-cultural contexts but who were from the same health care profession.

### Instrumentation

The BARRIERS Scale was used to measure participants’ perceived barriers to research utilization. This scale has been widely used with nurses [4, 30] and to a lesser extent, allied health professionals [3, 12]. The BARRIERS Scale contains four subscales derived from confirmatory factor analysis: (1) the *Adopter* (values, skills, and awareness); (2) the *Organization* (setting); (3) the *Innovation* (qualities of the research); and (4) *Communication* (presentation and accessibility of the research). The 28 items are rated according to the degree to which the respondent perceives the item to be a research barrier, rated from 1 (‘to no extent’), to 4 (‘to a great extent’). The “no opinion” response option was not provided in the current study (see discussion for further comments). The authors reported good internal consistencies of the first three factors (Cronbach’s α of 0.72–0.80), lower internal consistency for the fourth factor (Cronbach’s α of 0.65), and preliminary evidence of test–retest reliability with Pearson correlations ranging from 0.68 to 0.72 over a one week interval [10]. The BARRIERS Scale can be accessed at http://barriers.web.unc.edu/ or from the Funk et al. article [10]. The BARRIERS Scale has been widely used in many studies investigating barriers to research utilization [16] and has been translated previously into Turkish, German, Thai, Korean, and French. Recently the BARRIERS Scale was used to investigate barriers to EBP and research utilization with occupational therapists in Sweden, although it should be noted that no attempt at validating the structure of the instrument was undertaken [21].

For the version of the BARRIERS Scale used in this study, the word ‘nurse’ was replaced with ‘clinician’ as the participant group were occupational therapists. This did not alter the meaning or relevance of the BARRIERS Scale items. The version of the BARRIERS Scale completed by the participants in Taiwan was in Mandarin Chinese. It was translated from English to Mandarin Chinese by a qualified translator. The Mandarin Chinese version of the BARRIERS Scale was then reviewed by a panel of three bilingual (Mandarin Chinese–English) occupational therapists for phrasing, diction, understanding, and coherence. No changes to the scale were suggested by the panel. The Mandarin Chinese version of the BARRIERS Scale was then back-translated into English by another qualified translator. The back-translated English version of the BARRIERS Scale was then compared to the original version, in accordance with the translation procedures described by Cha et al. [31] and Wang et al. [32]. The items from the two versions of the BARRIERS Scale (English version and Taiwanese version) were similar in meaning, content, and wording.

### Data collection

Survey packages were mailed to prospective participants who met the inclusion criteria, and a reminder letter was sent 2 weeks following the initial survey distribution, as a method to facilitate an increased response rate. Survey responses were recorded anonymously since participants were not asked to report any personally identifying information and consent was inferred through completion of the questionnaire. Entry of the data obtained from respondents in Taiwan was completed in Taiwan while data entry for the Australian and United Kingdom data was completed in Australia. All surveys that were returned were complete, so missing data or incomplete questionnaires were not an issue.

### Statistical analysis

The BARRIERS scale was subjected to principal components analysis with Varimax rotation using Statistical Package for Social Sciences (SPSS), IBM version 20. To evaluate the dimensional structure and stability across samples, a Procrustes transformation was employed [33] using Orthosim version 2.01 [34]. Items were systematically removed based on the following criteria: (1) low communality (<0.3); (2) significant cross-loading (difference between primary and secondary loadings <0.3) [35]; and (3) primary loading on a factor inconsistent with the original theorized model. Less than ideal item Procrustes congruence values across all three participant groups was used to evaluate the cross-cultural equivalence, and the model fit comparisons were evaluated using overall congruence coefficient values.

## Results

### Participant demographics

Questionnaires were mailed to a total of 1230 participants (300 in Australia, 480 in the United Kingdom, and 450 in Taiwan). Responses were received from 696 participants (56.58 %), with a greater number of respondents from Taiwan (n = 413, response rate: 91 %), compared to Australia (n = 137, response rate: 45 %) or the United Kingdom (n = 144, response rate: 30 %). It should be noted that the response rate from Taiwan was notably higher compared to that of Australian and United Kingdom sample groups. This was likely to be as a result of attitudinal differences between the participant groups. Previous research has indicated that participants from different cultural backgrounds often have different attitudes towards voluntary participation in research studies [36, 37]. Taiwan is considered to be a collectivist culture [38], which is associated with higher levels of compliance than in individualistic cultures [39] such as Australia and the United Kingdom.

The sample consisted of 519 female and 177 male participants, with the majority aged between 20 and 39 years (77 %). Most of the participants reported their highest level of occupational therapy qualification as a bachelor’s degree (78 %), were working full-time (86 %), and held the position of an occupational therapy clinician for an employer (81 %). The majority of respondents reported their primary area of responsibility as clinical (87 %), had a caseload of outpatient/community based clients (41 %), and reported being employed in a general hospital setting (42 %) (see Table 1).

**Table 1:** Participant demographic data (*n* = 696)

	Frequency^a^	Percentage
Gender		
Female	519	74.60
Male	177	25.43
Total	696	100.03
Age (years)		
20–29 years	267	38.36
30–39 years	268	38.51
40–49 years	97	13.94
50–59 years	52	7.50
60+ years	5	0.72
Total	689	99.03
Country of residence		
Australia (response rate 45 %)	137	19.68
United Kingdom (response rate 30 %)	144	20.70
Taiwan (response rate 91 %)	413	59.34
Other	2	0.29
Total	696	100.01
Highest level of occupational therapy (OT) qualification obtained		
Diploma/certificate in OT	79	11.35
Bachelors degree in OT	540	77.59
Entry level masters degree in OT	8	1.15
Course work/research masters in OT	58	8.33
Research doctorate in OT	10	1.44
Total	695	99.86
Employment status		
Full-time between 20 and 40 h per week	594	85.34
Part-time less than 20 h per week	69	9.91
Student/postgraduate student	12	1.72
Non-practicing	10	1.44
Other	10	1.44
Total	695	99.86
Current position		
OT clinician working for an employer	563	80.90
Private practitioner	46	6.61
OT manager/administrator	36	5.17
Academic faculty/educator	14	2.01
Researcher	7	1.01
Consultant	2	0.29
Administrative coordinator/supervisor/case-manager	9	1.30
Other	18	2.59
Total	695	99.88

^a^The total number of participants included in the study was 696. However the ‘total’ categories do vary as some participants left some of the demographic questions blank

### Initial model analysis

Since confirmatory factor analysis is often considered overly stringent for exploratory investigations of data, [40] principal components analysis with Procrustes transformation was used [33]. This method compares the rotated solution to an ideal matrix where items either load completely or not at all; providing an estimate of how well items fit. For consistency and ease of presentation, the labels of the BARRIERS Scale items have been drawn from the original scale names. Items were numbered in the order presented in the original publication of the BARRIERS Scale [10].

The 28 items of the BARRIERS Scale [10] were subjected to Principal Components Analysis with Varimax rotation for each sample. Kaiser–Meyer–Olkin Measure of Sampling Adequacy values ranged from 0.789 to 0.966, and Bartlett’s Test of Sphericity was significant, supporting the factorability of the correlation matrix for each population [41–43].

Up to eight dimensions with eigenvalues exceeding 1 were found across the participant groups. However Cattell’s scree test [44] and Parallel Analysis [45] generally provided support for a four factor model, although there was slight support for a three component model in the United Kingdom sample and some support for a two component model for the Taiwanese sample. A forced four factor PCA with Varimax rotation was conducted for each sample, and a Procrustes transformation was performed using Orthosim version 2.01 [34]. The results of the initial model are presented in Table 2.

**Table 2:** Initial PCA loadings with Procrustes transformation by country

Item	Australia				United Kingdom				Taiwan				Procrustes congruence		
	1	2	3	4	1	2	3	4	1	2	3	4	AUS	UK	TAI
ADOPT1	*0.76*	0.19	0.09	−0.03	*0.73*	0.27	0.12	0.34	*0.79*	0.22	0.18	0.27	0.96	0.88	0.89
ADOPT2	*0.81*	0.26	0.06	0.07	*0.73*	0.22	0.04	0.39	*0.76*	0.22	0.22	0.26	0.96	0.90	0.87
ADOPT3	*0.68*	0.30	0.13	−0.07	*0.70*	0.20	0.22	0.29	*0.81*	0.25	0.23	0.18	0.90	0.85	0.88
ADOPT4	*0.48*	−0.17	0.04	0.11	*0.58*	−0.02	−0.09	−0.16	*0.48*	0.07	0.26	0.23	0.89	0.89	0.79^a^
ADOPT5	*0.71*	0.25	0.12	0.06	*0.71*	0.35	0.14	0.26	*0.75*	0.23	0.28	0.31	0.94	0.84	0.83
ADOPT6	0.36	*0.38*	0.02	0.23	0.36	0.39	−0.21	*0.47*	*0.55*	0.42	0.06	0.32	0.71^a^	0.66^a^	0.71^a^
ADOPT7	0.19	*0.46*	−0.26	0.20	0.19	*0.64*	0.09	0.15	*0.54*	0.54	0.20	0.16	0.45^a^	0.30^a^	0.63^a^
ADOPT8	0.34	*0.35*	0.00	0.25	0.47	*0.55*	0.16	0.15	0.50	*0.54*	0.24	0.21	0.70^a^	0.61^a^	0.59^a^
ORGAN1	0.06	*0.73*	0.12	0.01	*0.58*	0.40	0.11	−0.06	*0.56*	0.50	0.28	0.17	0.98	0.63^a^	0.69^a^
ORGAN2	0.07	*0.72*	0.17	−0.05	*0.50*	0.48	0.29	−0.02	*0.59*	0.42	0.35	0.20	0.97	0.74^a^	0.60^a^
ORGAN3	0.08	*0.47*	0.17	0.12	0.00	*0.80*	0.12	0.13	0.17	*0.78*	0.12	0.20	0.92	0.95	0.95
ORGAN4	0.22	*0.63*	−0.06	0.13	0.45	*0.50*	0.36	−0.08	*0.58*	0.48	0.31	0.19	0.89	0.78^a^	0.65^a^
ORGAN5	0.17	*0.68*	0.19	0.00	0.15	*0.71*	0.20	0.03	0.37	*0.68*	0.27	0.16	0.93	0.97	0.87
ORGAN6	0.09	*0.72*	0.17	−0.05	0.37	*0.56*	0.24	0.16	0.50	*0.58*	0.26	0.19	0.96	0.81	0.76^a^
ORGAN7	0.21	0.02	0.05	*0.40*	0.19	*0.58*	0.01	0.37	0.12	*0.69*	0.10	0.40	0.05^a^	0.73^a^	0.85
ORGAN8	0.33	*0.29*	0.13	0.26	0.42	*0.44*	0.35	0.29	0.36	*0.45*	0.40	0.38	0.53^a^	0.64^a^	0.65
INNOV1	0.20	−0.03	*0.67*	0.08	0.19	0.09	*0.51*	0.41	0.22	0.19	*0.77*	0.28	0.97	0.79^a^	0.87
INNOV2	0.17	0.08	*0.75*	0.14	0.33	0.07	*0.66*	0.39	0.41	0.18	*0.72*	0.33	0.96	0.85	0.80
INNOV3	0.08	0.09	*0.74*	0.11	−0.06	0.23	*0.78*	0.26	0.26	0.22	*0.76*	0.32	0.97	0.83	0.85
INNOV4	−0.20	0.19	*0.72*	−0.03	−0.02	0.20	*0.78*	0.22	0.22	0.28	*0.72*	0.34	0.89	0.86	0.81
INNOV5	0.15	0.18	*0.63*	0.26	0.18	0.17	*0.61*	0.32	0.41	0.28	*0.53*	0.42	0.87	0.83	0.63^a^
INNOV6	0.04	0.08	0.16	*0.41*	0.32	−0.07	0.22	*0.43*	0.34	−0.05	0.42	*0.53*	0.34^a^	0.56^a^	0.64^a^
COMM1	−0.03	0.30	0.33	*0.47*	−0.01	0.23	0.28	*0.62*	0.28	0.22	0.32	*0.64*	0.72^a^	0.87	0.76^a^
COMM2	−0.06	−0.05	−0.01	*0.70*	0.10	0.10	0.20	*0.51*	0.30	0.23	0.25	*0.71*	1.00	0.85	0.80
COMM3	0.08	0.11	0.38	*0.66*	0.11	0.10	0.29	*0.62*	0.28	0.33	0.31	*0.69*	0.84	0.83	0.75^a^
COMM4	0.14	0.03	0.13	*0.61*	0.01	0.08	0.15	*0.65*	0.13	0.33	0.18	*0.68*	0.93	0.94	0.85
COMM5	−0.05	0.02	−0.05	*0.68*	0.05	0.07	0.16	*0.63*	0.17	0.18	0.26	*0.72*	0.99	0.92	0.86
COMM6	0.13	0.28	*0.42*	0.29	0.23	0.22	0.28	*0.28*	0.45	0.20	0.27	*0.57*	0.46^a^	0.49^a^	0.66^a^

Major loadings are in italics

*ADOPT* Characteristics of the adopter: the clinician’s research values, skills, and awareness’, *ORGAN* characteristics of the organisation: setting barriers and limitations, *INNOV* characteristics of the innovation: qualities of the research, *COMM* characteristics of the communication: presentation and accessibility of the research

^a^Lower than ideal congruence coefficient

From Table 2, it can be seen that a considerable number of items demonstrated a primary loading on components other than those originally defined by Funk et al. [10] Item cross-loading is evident for many items and across all three participant groups. The Procrustes transformation provides an indication of item fit to an ideal matrix, with values below 0.80 suggesting less than ideal item fit [46]. Overall model fit was also calculated (Australia = 0.81; United Kingdom = 0.78; Taiwan = 0.77), with values below 0.85 indicative of less than ideal model fit [47, 48].

### Final model analysis

The final model which provided the best overall fit is described in Table 3.

**Table 3:** Final PCA loadings with procrustes transformation by country

Item	Australia				United Kingdom				Taiwan				Procrustes congruence		
	*1*	2	3	4	1	2	3	4	1	2	3	4	AUS	UK	TAI
ADOPT1	*0.79*	0.15	0.07	0.01	*0.80*	0.32	0.14	0.18	*0.83*	0.21	0.21	0.22	0.97	0.85	0.84
ADOPT2	*0.86*	0.19	0.06	0.08	*0.80*	0.26	0.11	0.20	*0.80*	0.21	0.24	0.23	0.96	0.88	0.82
ADOPT3	*0.75*	0.25	0.12	−0.03	*0.78*	0.25	0.25	0.06	*0.81*	0.26	0.25	0.16	0.93	0.89	0.81
ADOPT5	*0.72*	0.20	0.14	0.05	*0.71*	0.43	0.17	0.07	*0.74*	0.25	0.32	0.26	0.93	0.81	0.75^a^
ORGAN1	0.11	*0.73*	0.08	0.01	0.32	*0.64*	−0.16	0.29	*0.54*	0.53	0.26	0.21	0.99	0.87	0.78^a^
ORGAN2	0.13	*0.68*	0.16	−0.09	0.32	*0.69*	0.04	0.18	0.46	*0.60*	0.34	0.23	0.95	0.91	0.81
ORGAN3	0.07	*0.57*	0.11	0.16	0.06	*0.67*	0.24	−0.03	0.14	*0.80*	0.13	0.23	0.95	0.90	0.96
ORGAN4	0.26	*0.61*	−0.06	0.14	0.25	*0.71*	0.14	0.12	0.54	*0.55*	0.28	0.23	0.91	0.92	0.78^a^
ORGAN5	0.21	*0.73*	0.13	0.01	0.10	*0.73*	0.19	−0.01	0.31	*0.76*	0.26	0.18	0.96	0.94	0.93
ORGAN6	0.13	*0.76*	0.12	−0.03	0.35	*0.63*	0.25	−0.01	0.46	*0.66*	0.24	0.21	0.98	0.81	0.87
INNOV1	0.15	−0.01	*0.70*	0.03	0.22	0.14	*0.54*	0.34	0.22	0.17	*0.78*	0.26	0.97	0.80	0.91
INNOV2	0.15	0.09	*0.77*	0.08	0.29	0.21	*0.58*	0.43	0.42	0.17	*0.75*	0.28	0.97	0.74^a^	0.83
INNOV3	0.10	0.09	*0.77*	0.08	−0.07	0.27	*0.80*	0.15	0.25	0.21	*0.79*	0.26	0.98	0.95	0.89
INNOV4	−0.20	0.25	*0.70*	−0.03	−0.04	0.28	*0.78*	0.14	0.21	0.26	*0.76*	0.31	0.91	0.95	0.87
INNOV5	0.15	0.16	*0.67*	0.24	0.28	0.23	*0.71*	−0.04	0.40	0.29	*0.57*	0.37	0.89	0.90	0.70^a^
COMM2	−0.05	0.00	−0.02	*0.71*	0.08	0.09	0.14	*0.83*	0.33	0.16	0.30	*0.69*	1.00	0.96	0.80
COMM3	0.11	0.11	0.41	*0.67*	0.17	0.07	0.36	*0.67*	0.30	0.27	0.36	*0.69*	0.85	0.86	0.76^a^
COMM4	0.16	0.01	0.16	*0.69*	0.38	−0.16	0.08	*0.56*	0.13	0.25	0.21	*0.76*	0.96	0.89	0.89
COMM5	−0.06	0.05	0.00	*0.71*	0.28	−0.03	0.26	*0.46*	0.18	0.15	0.29	*0.77*	0.99	0.82	0.88

Major loadings are in italics

*ADOPT* characteristics of the adopter: the clinician’s research values, skills, and awareness, *ORGAN* characteristics of the organisation: setting barriers and limitations, *INNOV* characteristics of the innovation: qualities of the research, *COMM* characteristics of the communication: presentation and accessibility of the research

^a^Lower than ideal congruence coefficient

**Fig. 1:**
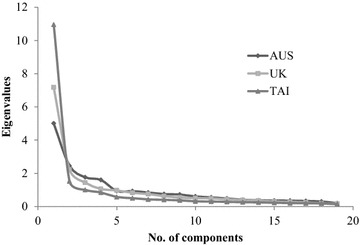
Scree plot for components by country

For each the three participant group, the Kaiser–Meyer–Olkin Measure of Sampling Adequacy values ranged from 0.797 to 0.958. Additionally, the Bartlett’s Test of Sphericity was significant, supporting the factorability of the correlation matrix [41–43]. Similarly to the initial model, a four component solution was more strongly supported in the Australian sample, while there was slight support for a three component solution for the United Kingdom sample, and some support for a two component solution for the Taiwan sample. While this could be indicative of lower discriminant validity in the later samples, it is also likely to occur if the response styles are similar across items within countries. Figure 1 details the scree plots by country.

For the Australian participants, the four factor model explained 26.36, 12.95, 9.27 and 8.43 % of the variance. For the British participants, the four factor model explained 37.79, 11.71, 7.56 and 5.57 % of the variance. For Taiwanese participants, the four factor model explained 57.67, 8.04, 5.29 and 4.44 % of the variance. From Table 3, it is evident that across all populations, the issues identified in the initial model are greatly reduced. Overall model fit was also significantly improved, with the Procrustes model fit congruence coefficients for Australia, United Kingdom and Taiwan calculated to be 0.95, 0.88, and 0.85 respectively.

An alternative application for the Procrustes transformation procedure is the comparison of component loadings between samples, rather than to an ideal matrix. This provides an indication of how well the component structure of one sample compares to another. The cross-cultural comparison demonstrated excellent results, with Procrustes model fit congruence coefficients for Australia–United Kingdom, Australia Taiwan, and United Kingdom–Taiwan calculated to be 0.93, 0.92, and 0.94 respectively. Despite the support for two or three component solutions in the Taiwan and United Kingdom samples respectively, the cross-cultural congruence coefficients provide support for the interpretability of the four component solution across cultures. Item level congruence coefficients are detailed in Table 4, while the content of the final 19 items can be found in Table 5.

**Table 4:** Cross-cultural procrustes congruence coefficients

Item	Australia–United Kingdom	Australia–Taiwan	United Kingdom–Taiwan
ADOPT1	0.94	0.94	1.00
ADOPT2	0.97	0.94	0.98
ADOPT3	0.97	0.96	0.98
ADOPT5	0.96	0.93	0.98
ORGAN1	0.88	0.86	0.86
ORGAN2	0.93	0.89	0.95
ORGAN3	0.89	0.99	0.90
ORGAN4	0.94	0.89	0.95
ORGAN5	0.98	0.98	0.96
ORGAN6	0.92	0.93	0.96
INNOV1	0.86	0.94	0.96
INNOV2	0.87	0.95	0.95
INNOV3	0.96	0.97	0.94
INNOV4	0.97	0.86	0.95
INNOV5	0.91	0.93	0.86
COMM2	0.95	0.74^a^	0.89
COMM3	1.00	0.95	0.95
COMM4	0.96	0.93	0.81
COMM5	0.77^a^	0.86	0.95

^a^Less than ideal congruence coefficient

**Table 5:** Statements for 19 item BARRIERS Scale

Item code	Item content
ADOPT1	The clinician does not see the value of research for practice
ADOPT2	The clinician sees little benefit for self
ADOPT3	The clinician is unwilling to change/try new ideas
ADOPT5	The clinical feels the benefits of changing practice will be minimal
ORGAN1	Administration will not allow implementation
ORGAN2	Physicians will not cooperate with implementation
ORGAN3	There is insufficient time on the job to implement new ideas
ORGAN4	Other staff are not supportive of implementation
ORGAN5	The facilities are inadequate for implementation
ORGAN6	The clinician does not feel she/he has enough authority to change client care procedures
INNOV1	The research has methodological inadequacies
INNOV2	The conclusion drawn from the research is not justified
INNOV3	The research has not been replicated
INNOV4	The literature reports conflicting results
INNOV5	The clinician is uncertain whether to believe the results of the research
COMM2	Research reports/articles are not readily available
COMM3	The research is not reported clearly and readably
COMM4	Statistical analyses are not understandable
COMM5	The relevant literature is not compiled in one place

Items adapted from Funk et al. [10]

### Validity and reliability of the final model

To investigate the validity of the scales in the final model, Pearson’s correlations were calculated for scale scores calculated using the original structure, and the adapted final structure. The resulting correlations were all highly significant, indicating that the scales in the final model are comparable to those used in the original model; and as such, interpretation of the final model scales and comparison to previously published studies will be meaningful. The correlations are reported in Table 6.

**Table 6:** Cronbach’s α reliability coefficients for adapted scales by country

Adapted scale	Australia	United Kingdom	Taiwan
ADOPT	0.80	0.90	0.92
ORGAN	0.80	0.83	0.91
INNOV	0.79	0.85	0.92
COMM	0.68	0.66	0.86

Reliabilities for the adapted final model scales were calculated using Cronbach’s alpha. All scales indicated good or better reliability across samples with the exception of the *Communication* subscale, which demonstrated lower (although acceptable) reliability in the Australian and United Kingdom populations. Scale reliabilities are detailed in Table 7.

**Table 7:** Pearson’s correlation for original and adapted scales by country

	Adapted scales		
	Australia	United Kingdom	Taiwan
Original			
ADOPT	0.91	0.95	0.95
ORGAN	0.96	0.98	0.98
INNOV	0.96	0.98	0.99
COMM	0.93	0.94	0.97

All correlations were significant *p* < 0.001

## Discussion

The purpose of the study was to investigate the dimensional structure and stability of the BARRIERS Scale across three cross-cultural cohorts of occupational therapy clinicians. The findings indicate that the 19 item, four component solution provided a superior fit for structure and dimensionality compared to the original 28 item solution when considered across cultures. It also provided evidence of the component stability when compared between the three participant groups. The final model obtained in this study largely mirrored the original factor structure obtained by Funk et al. [10] which was supported by the high correlations between the original scale scores and final adapted scale scores. The primary difference between the original BARRIERS Scale version and the final model version obtained derived in this study was the number of items with the new version only being composed of 19 of the original items. The final model also demonstrated internal reliability equal to or better than that published by Funk et al. [10, 13].

The current study included a slight change to the administration of the BARRIERS Scale, with the removal of the “no opinion” response. Endorsement of not having an opinion acts as a confounder to meaningful statistical analysis. Missing value replacement protocols rely on an assumption of data missing at random or completely at random [35] which is not able to be adequately established. Previous studies which have investigated the dimensional validity of the BARRIERS Scale have chosen to either to exclude those cases (leading to a severe loss of sample size) [10] or substitute for the first response “to no extent” [12]. However to do the later suggests that having “no opinion” is equivalent to a barrier impacting on the participant “to no extent”, and that this assumption holds across all participants—an assumption that is optimistic at best, and seriously flawed at worst.

In an Australian study, Hutchinson and Johnston reported achieving an acceptable four factor solution using 27 items of the BARRIERS Scale [14]; however an investigation of the solution revealed a number of items which had primary loadings on incorrect factors, and other items with significant cross-loading. This is consistent with the initial model in the current study, and was the rationale for conducting further item removal. Similarly, in a study conducted in the United Kingdom, the factor structure of the original scale did not emerge, and the results suffered from poor validity and reliability. [27] A later study which focused on revised items fared little better. [9] These results were also consistent with the initial results found in the United Kingdom sample in the current study, where items were loading on incorrect factors and there were a considerable number of items with poor congruence coefficients.

No previous research was identified that described the use of the BARRIERS Scale in Taiwan, or where the BARRIERS Scale had been translated into Mandarin Chinese. Previous translations have been applied in countries such as Korea [19] and Iran [49]. The rigorous translation and back-translation procedure as described by Cha et al. [31] and Wang et al. [32] preserved the meaning and content of the BARRIERS Scale items, with the final model demonstrating acceptable fit. While several items demonstrated less than ideal fit in the Taiwanese participant group, these items proved to be not problematic in the overall fit. One item demonstrated a primary loading on the incorrect factor; however the difference between the primary and secondary loading was only 0.01.

To achieve acceptable model fit across the three samples, a total of nine items were removed. Three criteria were used to determine which items to remove, including low communalities, significant cross-loading, and items which demonstrated a primary loading on a component other than as originally hypothesised. Items with low communality are problematic, suggesting that the items offer little information of use while confounding the desired constructs. Significant cross-loading of items suggests a lack of discriminant validity, which can lead to inflated correlations between constructs [35]. Items with a primary loading on the incorrect component are not consistent with the theoretical underpinnings of the scale and removal of such items is worthy of consideration. While it could be argued that item removal is a threat to the depth of each construct, if the removal results in an increase in reliability, discriminant validity, and dimensional stability across samples and cultures, then the item removals are warranted.

Within the *Adopter* subscale, four items were removed. ADOPT7 (*The clinician is isolated from knowledgeable colleagues with whom to discuss the research*) and ADOPT8 (*The clinician is unaware of the research*) demonstrated low communality in the Australian sample, cross-loading across all samples, and a primary loading on the *Organisation* subscale across all samples, suggesting that these items offer little discriminant validity and may be more indicative of organisational barriers to research utilisation—albeit poorly performing ones. ADOPT6 (*The clinician does not feel capable of evaluating the quality of the research*) demonstrated cross-loading in all samples and primary loadings on the *Organisation* and *Communication* subscales for the United Kingdom and Taiwan samples respectively, suggesting that this item offered little discrimination. ADOPT4 (*There is not a documented need to change practice*) demonstrated low communality in the Australian sample, which may have been indicative of method error, given that all other items in *Adoption* were worded from the perspective of “the clinician” which was noticeably absent in this item.

Within the *Organisation* subscale, two items were removed. ORGAN7 (*The clinician does not have time to read research*) and ORGAN8 (*The clinician feels results are not generalisable to own setting*) demonstrated low communalities in the Australian sample and primary loadings on the *Communication* and *Adopter* subscales respectively. While this was not evident in the other samples, significant cross-loading was noted, suggesting that while not as problematic in the United Kingdom and Taiwan samples as the Australian sample, nevertheless the items were a threat to cross-cultural comparisons.

Within the *Innovation* subscale, only one item was removed. INNOV6 (*Research reports/articles are not published fast enough*) demonstrated low communality in the Australian sample, and primary loading on the *Communication* subscale across all samples. Semantically, the relationship between the timely availability of research and accessibility of research appears to be meaningful, however given that the item was not hypothesised to load on this component, removal was justified.

Within the *Communication* subscale, two items were removed. COMM1 (*Implications for practice are not made clear*) demonstrated significant cross-loading in the Australian sample. COMM6 (*The research is not relevant to the clinician’s practice*) performed rather inconsistently across samples, loading on *Innovation* in the Australian sample, demonstrating low communality in the United Kingdom sample, and cross-loading in the Taiwan sample.

Cross-cultural comparisons using Procrustes transformation allowed for further evaluation of the structural integrity and dimensionality of the 19 item BARRIERS Scale. While overall fit was excellent, two items indicated less than ideal fit across populations. Between Australia and Taiwan, the fit for COMM2 (*Research reports/articles are not readily available*) was less than ideal, and a review of the component loadings suggested that this was mostly as a result of larger secondary loadings in the Taiwanese sample, with the primary loadings differing between participant groups by only 0.02. Between Australia and the United Kingdom, COMM5 (*The relevant literature is not compiled in one place*) also indicated misfit, with a lower primary loading and cross-loading in the United Kingdom sample. This pattern may indicate that the availability of relevant literature is less of a concern in the United Kingdom than in Australia.

The *Communication* subscale demonstrated lower reliability in both the original study [10] and in the final model of the current study. This indicates that the *Communication* subscale has been consistently underperforming when compared to the other BARRIERS subscales, and research may be warranted in further refining this subscale through the development of additional items. Interestingly, the *Communication* subscale demonstrated much higher reliability in the Taiwanese sample, which suggests that the content of the items in the final model of the subscale may be more indicative of barriers to research relating to presentation and accessibility in Taiwan than in Australia or the United Kingdom.

A brief comment is warranted on the variance explained by the 19 item four component model across samples. The total variance explained for Australia, United Kingdom, and Taiwan was 57.01, 62.63, and 75.44 % respectively. Some explanation can be found in the scree plots (see Fig. 1). While a four component solution was clearly demonstrated for the Australian sample, there was a slight inclination towards a three component solution for the United Kingdom sample, and some evidence for a two component solution for the Taiwan sample. This suggests that there may be somewhat less discriminant validity between the components in the United Kingdom and Taiwan samples, or an element of “blurred boundaries” between the components [50]. As more components are extracted, progressively more variance is explained which contributes to the higher total variance reported in these later samples. When the forced four component model was compared cross-culturally, the slightly reduced discriminant validity was not problematic given the excellent model fit, supporting the use of the four component model in the cross-cultural comparison. Of the 13 studies identified by Kajermo et al. [17] which investigated the structure of the BARRIERS scale, eight reported values for total variance explained. Interestingly, the mean variance explained across studies was only 44.03 % (range 38.90–47.50 %), which provides further support for the item removal process in the current study given the increase in variance explained by the 19 item model.

### Study limitations

The study has several potential limitations. Firstly, the use of self-report data has a number of bias issues such as answering scale items in a socially desirability manner. Secondly, the use of convenience sampling, while easier to recruit participants, can limit the generalisability of results, although three different sample groups strengthen the external validity. The current study also used relatively small sample sizes for Australia and the United Kingdom, although these proved to be not problematic. Response rates varied across samples, however this may be somewhat explained by cultural differences in compliance [37].

### Future research

Several suggestions for future research are apparent. The consistent lower reliability of the *Communication* subscale suggests that further research is warranted to more accurately identify and measure these barriers to research. The final model presented in the current study requires fewer items while preserving the meaning of the original BARRIERS Scale factor structure, and demonstrated cross-cultural structural and dimensional stability. The rigorous psychometric evaluation presented in the current study allows for meaningful comparisons to be drawn between paediatric occupational therapists in Australia, the United Kingdom, and Taiwan. While not explicitly tested in the current study, it would be worth exploring the extent to which the same dimensional stability is evident across other cultures and professions, to assist in informing evidence based practice. It is also suggested that the 19 item, four component version of the BARRIERS Scale be used by clinicians who might be interested in identifying key self-reported barriers to research utilization and evidence based practice in health care settings.

## Conclusion

The 19 item version of the BARRIERS Scale is a robust, valid and reliable measure. It appears to have applicability in cross-cultural settings and also has decreased respondent burden since it has nine fewer items compared to the original 28 item version. The 19 item version of the BARRIERS Scale demonstrated acceptable dimensional stability when completed by participants from three different cross-cultural contexts. Further evaluation of the measurement properties of the 19 item BARRIERS Scale across health professions is recommended.
